# Early Life Influences Kidney Function at Age 63–64 Years, but So Does Adult Body Size: Results from the Newcastle Thousand Families Birth Cohort

**DOI:** 10.1371/journal.pone.0066660

**Published:** 2013-06-13

**Authors:** Stephanie L. Harrison, Kay D. Mann, Mark S. Pearce

**Affiliations:** 1 School of Biomedical Sciences, Newcastle University, Newcastle upon Tyne, United Kingdom; 2 Institute of Health & Society, Newcastle University, Newcastle upon Tyne, United Kingdom; Mario Negri Institute for Pharmacological Research and Azienda Ospedaliera Ospedali Riuniti di Bergamo, Italy

## Abstract

**Background:**

It is suggested that impaired fetal growth can affect kidney development, resulting in fewer glomeruli being formed and reduced kidney function later in life. The aim of this study was to investigate early life variables in relation to adult kidney function, and compare these to the influence of later life variables.

**Methods:**

Detailed information was collected prospectively regarding 1,142 babies, born in 1947 in Newcastle upon Tyne. At the age of 63–64 years, 335 participants had serum creatinine successfully measured and completed a lifestyle questionnaire. These measurements were used to calculate their estimated glomerular filtration rate (eGFR).

**Results:**

Body mass index (BMI) and being female were significantly negatively associated with eGFR. Birth weight was significantly positively associated with eGFR. In sex-specific analyses, BMI and cigarette smoking remained significant for males (n = 154), with a near significant association for birth weight, whereas none of the variables remained significant for females (n = 181).

**Conclusions:**

The findings suggest that sex, size at birth and BMI may be important variables influencing adult kidney function. However, as only a small amount of variance in eGFR was explained by these variables, additional longitudinal studies would be beneficial for assessing lifecourse influences on kidney function.

## Introduction

It has been estimated that 1 in 10 people in the United States [1] and the United Kingdom [2] are affected by chronic kidney disease, with consequences for both the individual and healthcare services. During fetal development, nephrogenesis occurs from as early as 5 weeks in gestation. However, the rate of nephrogenesis increases rapidly from around the middle of the second trimester (week 21), and finishes at week 36 [3]. Therefore, this developmental stage is crucial to kidney function, and animal studies have shown various factors can influence this stage, such as foetal nutrition [4], [5]. Factors affecting the normal progression of nephrogenesis during this “critical window” in foetal growth can change the structure of the kidney [6], the number of nephrons a person is born with [4], [5] and can potentially lead to alterations in adult life kidney function [7].

A number of studies have found a positive association between birth weight and eGFR [8]–[12], but others have found no association [13]–[15]. However, the majority of these studies were conducted in children or young adults, and the oldest mean age for any of these studies was 49 years. The age at which these studies were conducted is important, as the effect of early life influences on kidney function may not become apparent until later in adult life.

Further, little research has been done to investigate the impact of other early life influences in relation to adult kidney function such as socio-economic status (SES) and breastfeeding. Research into the associations between later life influences and kidney function have shown conflicting results for body mass index (BMI) [16]–[20] and smoking [21]–[25], and a positive association for current SES [26]–[28]. The Newcastle Thousand Families birth cohort [29]–[30] provided a unique opportunity to analyse longitudinal data and investigate the effects of both early and later life influences on kidney function at age 63–64 years.

## Methods

### Ethics statement

The study received ethical approval from the Sunderland Local Research Ethics Committee and all study members gave their written consent.

The Newcastle Thousand Families study began as a prospective study of all 1142 children born in May and June 1947 to mothers resident in Newcastle upon Tyne, in northern England [29]–[30]. The health, growth and development of the cohort were followed in great detail up to age 15 years. Throughout the first years of the children's lives, all families were visited both on a routine (up to every six weeks during infancy and at least quarterly until age five years) and on an *ad hoc* basis by the study team, which consisted of health visitors (nurses who visited families at home) and paediatricians.

The cohort underwent a major follow-up at age 49–51 years [29], and again at age 63–64 years. In both follow-up waves, health and lifestyle questionnaires were sent out for completion and return and study members were invited to attend for clinical examination. The latest follow-up wave was conducted between 2010 and 2011, with 354 of the original 1142 participants (31.0%) returning for a clinical examination. There have been 183 known deaths in the cohort, including 2 from renal disease (one in 2002, and one in 2011 who did not participate in the latest follow-up wave).

Information on early life was recorded prospectively for all study members [29]–[30]. Birth weights, as recorded by the midwife at the time of the child's birth, were standardised for gestational age and sex [31]. Socioeconomic status at birth (I to V, with I assumed to be the most advantaged and V the least advantaged) was measured by paternal occupational social class at the time of the child's birth. Housing conditions were assessed by the city's Public Health Department near the time of the child's birth, and scored for the presence of overcrowding, lack of hot water, toilets shared between households and dampness or poor repair. Duration of being breast fed was defined as the length of time a study member was at least partly breast fed, as recorded by the health visitors.

The number of cigarette pack-years smoked (one pack-year represents one pack of 20 cigarettes smoked per day for one year) were estimated from the responses to the questionnaires at age 49–51 years (which included additional questions about smoking habits at ages 15, 25 and 35) and 63–64 years. Four categories of alcohol consumption at age 49–51 years were derived from responses to the age 63–64 questionnaire: No drinking; light drinking (up to ten units/week of alcohol for males, 5 units for females); moderate drinking (11–28 units for males, 6–21 units for females) and heavy drinking (>28 units for males, >21 units for females). In the United Kingdom, one unit is 10 ml or 8 g of pure alcohol. SES at age 49–51 years was based on the occupation of the main wage earner in the household, according to the 1990 United Kingdom Registrar General's Standard Occupational Classification. This was used rather than at age 63–64 years by which time retirement or semi-retirement may make occupation-based measures of SES meaningless. The questionnaire included a section on self-reported medication use, the responses to which were used to identify participants taking angiotensin converting enzyme inhibitor (ACEI) medication, primarily due to high blood pressure.

### Clinical assessment

Height and weight were measured by research nurses, at the clinical assessment at age 63–64 years, and BMI derived. Kidney function was assessed by estimation of GFR, with low eGFR being indicative of poor kidney function, from the fasting serum creatinine measurements (μmol/L) recorded during the clinical assessment. Serum creatinine was divided by 88.4 to convert into mg/dL, in order to use in the GFR estimation which followed the Modification of Diet in Renal Disease equation [32]. All of the participants were Caucasian; therefore, the race section of the equation was irrelevant.

### Statistical analysis

SES, sex, housing grade and duration breastfed were treated as categorical variables. All other explanatory variables were treated as continuous linear variables. Representativeness of the participants in this study compared to the remainder of the original 1947 cohort was assessed using chi-squared tests for categorical variables and t-tests for continuous variables.

Univariate linear regression was initially used to test for associations between independent variables and the dependent variable, eGFR, followed by multivariable models for adjusted analyses. The coefficient of determination (R^2^) was estimated, to indicate how much variation in eGFR was due to the variables in the model. Interactions were tested within the regression modelling framework. Statistical analysis was done using the statistical software package, Stata version 11.0.

## Results

Serum creatinine was successfully recorded for 335 of the 354 participants. There were significant differences between the sample included in this study and the remainder of the original cohort for sex (p = 0.027) and SES at birth (p = 0.001). In the original cohort there were more males compared to females; however, more females compared to males returned for the recent follow-up study. The percentage of people who were born in SES 1 remained similar; however, this value was higher for SES 2 and lower for SES 3. No significant differences were found for the remainder of the variables tested (p>0.05).

Continuous data used in the study are summarised in table 1, while categorical data are summarised in table 2. Birth weights in the included participants ranged from 2.15 to 5.10 kg. Females were significantly lighter at birth than males (p = 0.021), but had significantly higher standardised birth weights (p = 0.004). Only 8 (3%) of participants were low birth weight (i.e. <2.5 kg). Only three of the included participants were premature births. Median eGFR was 78 mL/min/1.73 m^2^ (interquartile range (IQR) 22). Thirty six (11%) of included participants self-reported use of ACEI medication. This was not significantly associated with eGFR (p = 0.23).

**Table 1:** Descriptive statistics, continuous variables.

Variable	n	mean/median	std dev/IQR
**Estimated glomeral filtration rate (mL/min/1.73 m^2^)**	335	78.00	22.00
**Standardised birth weight**	326	−0.10	1.07
**Birth weight (kg)**	326	3.40	0.54
**Gestational age (weeks)**	326	40.00	0.00
**Cigarette pack-years smoked up to age 63–64 years**	315	0.20	21.20
**Body Mass Index age 63–64 years (kg/m^2^)**	335	27.90	4.97

**Table 2:** Descriptive statistics, categorical variables.

Variable	categories	n	%
**Sex**	Male	154	46
	Female	181	54
**Social class at birth**	I, II (most advantaged)	36	11
	IIIn, IIIm (middle)	210	65
	IV, V (least advantaged)	77	24
**Housing grade at birth**	0	160	49
	1	84	26
	2	47	15
	3 or more	34	10
**Duration breast fed**	Less than 4 weeks	76	24
	4 weeks to 6 months	153	47
	6 months and above	94	29
**Social class at age 50 years**	I, II (most advantaged)	161	58
	IIIn, IIIm (middle)	84	30
	IV, V (least advantaged)	34	12
**Smoking status at age 63–65 years**	Never smoked	147	45
	Ex smoker	152	46
	Current smoker	31	9
**Alcohol consumption at age 63–64 years**	None	34	10
	Light	134	41
	Moderate	121	37
	Heavy	38	12

Significant univariate associations with eGFR were seen for sex, birth weight, current smoking status, pack-years of cigarettes smoked, alcohol consumption and BMI (table 3), with sex, birth weight, pack-years and BMI significant in the multivariate analysis (table 4). Being female was associated with an estimated 3.83 mL/min/1.73 m^2^ reduction in eGFR in the adjusted model. Birth weight was positively associated with eGFR (p = 0.016), and a 1 kg increase in birth weight was associated with an estimated 3.85 mL/min/1.73 m^2^ increase in eGFR. There was no evidence of non-linearity in this association. However, there was not a significant association with birth weight when standardized for gestational age and sex or when comparing between low and normal birth weights. The number of cigarette pack-years smoked was positively associated with eGFR with each additional pack-year associated with an estimated increase in eGFR of 0.14 mL/min/1.73 m^2^. Contemporary BMI, was the most significant factor affecting eGFR independently (p = 0.001), and a 1 kg/m^2^ increase in BMI was associated with a 0.047 mL/min/1.73 m^2^ estimated decrease in eGFR. Adjusting for ACEI medication use made little difference to the results. The model containing the significant variables explained 8.7% of the variation in GFR in this cohort. There were no significant interactions for any of the variables tested, including between either birth weight or low birth weight and eGFR (p>0.05).

**Table 3:** Univariate linear regression results for eGFR at age 63–64 years.

Variable	Coefficient	95% CI	p-value	R^2^
**Sex**				
Male	Reference		0.010	0.020
Female	−4.75	−8.33,−1.16		
**Standardised birth weight**	1.10	−0.58, 2.79	0.198	0.198
**Birth weight (kg)**	4.13	0.78, 7.48	0.016	0.018
**Gestational age (weeks)**	1.52	−0.25, 3.28	0.093	0.009
**Social class at birth**				
I, II (most advantaged)	Reference		0.169	0.011
IIIn, IIIm (middle)	−4.54	−10.48, 1.40		
IV, V (least advantaged)	−6.38	−13.03, 0.27		
**Housing grade at birth**				
0	Reference		0.649	0.005
1	−1.85	−6.24, 2.54		
2	1.31	−4.10, 6.72		
3 or more	1.62	−4.54, 7.78		
**Duration breast fed**				
Less than 4 weeks	Reference		0.730	0.002
4 weeks to 6 months	−0.73	−5.35, 3.88		
6 months and above	1.00	−4.07, 6.08		
**Social class at age 50 years**				
I, II (most advantaged)	Reference		0.920	0.001
IIIn, IIIm (middle)	0.68	−3.80, 5.16		
IV, V (least advantaged)	1.10	−5.18, 7.37		
**Smoking status at age 63–64 years**				
Never smoked	Reference		0.009	0.029
Ex smoker	1.66	−2.13, 5.45		
Current smoker	10.24	3.76, 16.71		
**Cigarette pack-years smoked up to age 63–64 years**	0.14	0.03, 0.24	0.009	0.022
**Alcohol consumption at age 63–64 years**				
None	Reference		0.003	0.042
Light	−1.11	−7.35, 5.13		
Moderate	5.37	−0.94, 11.67		
Heavy	7.45	−0.22, 15.12		
**Body Mass Index age 63–64 years (kg/m^2^)**	−0.54	−0.90, −0.18	0.003	0.025

**Table 4:** Multivariable linear regression results for eGFR at age 63–64 years.

Variable		Coefficient	95% CI	P
**Sex**	Male	Reference		0.041
	Female	−3.83	−7.51, −0.16	
**Birth weight (kg)**		3.85	0.48, 7.23	0.025
**Cigarette pack-years smoked up to age 63–64 years**		0.14	0.03, 0.24	0.01
**Body Mass Index age 63–64 years (kg/m^2^)**		−0.65	−1.01, −0.28	0.001

In sex-specific analyses, BMI and cigarette smoking remained significant for males (n = 154), with a near significant association for birth weight (table 5). No variables were significantly associated with eGFR in the analysis restricted to females. Upon analysis for males exclusively BMI (R^2^ 6.0%) had a twofold increased contribution to eGFR in comparison to the contribution from birth weight (R^2^ 3.1%).

**Table 5:** Multivariable linear regression results for eGFR at age 63–64 years, restricted to males.

Variable	Coefficient	95% CI	P
**Birth weight (kg)**	5.06	−0.70,10.81	0.085
**Cigarette pack-years smoked up to age 63–64 years**	0.27	0.12,0.41	0.001
**Body Mass Index age 63–64 years (kg/m^2^)**	−1.19	−1.83,−0.55	<0.001

## Discussion

In this study of data from the Newcastle Thousand Families birth cohort, there were significant associations between eGFR and BMI, birth weight and pack-years of cigarettes smoked. However, these findings were limited to the males within the cohort, albeit of only borderline significance for birth weight; with no significant associations seen in females.

The result that a lower birth weight was significantly associated with a lower eGFR is consistent with the hypotheses put forward by Barker [33] and Brenner and Chertow [34], and with previous studies in humans [8]–[12]. Being born of a lower birth weight has been associated with a reduced number of glomeruli; the kidneys may overcompensate for this in the form of hyperfiltration, yet at a later stage of life this initially beneficial mechanism may have damaging effects. As the age of participants in the recent follow-up study was 63–64 years, the reduction in eGFR seen in those with lower birth weights could be a result of accelerated kidney damage, due to the initial hyperfiltration that Brenner and Chertow described [34]. This initial alteration of kidney development, during fetal growth, may account for the reduced eGFR seen in this study. However, no association was found between standardised birth weight, a better measure of fetal growth than birth weight alone, and eGFR, which is contrast to some previous studies in children [12] and young adults [9], although, these studies were at a much younger age than in this study.

Very few of the included study members had birth weights below 2.5 kg. This may make our findings more relevant to the entire birth weight range, rather than in studies focussing on comparisons between low and normal weight births, If the mechanism is through nephron number, this is consistent with the finding of a positive correlation between birth weight and number of nephrons, across the birth weight range [35]. The number of pre-term births in this study was very small. It is possible that a larger study with more pre-term births could see an association with prematurity and eGFR alongside, or in place of, an association with birth weight. We found different results when using crude and standardised birth weights. This is likely due to the influential adjustments for both gestational age and sex, which could be more important risk factors than birth weight alone.

The sex differences seen in this study are similar to previous reports from the cohort in terms of sex differences in lifecourse predictors of chronic disease biomarkers [36]–[37]. This is also consistent with some previous studies relating birth weight to previous kidney function [38]. With prospectively recorded birth weights, there is no possible recall bias between males and females. There is no clear biological explanation for differential effects, but it has been suggested that factors such as higher levels of oestrogen in females and earlier onset of diseases such as diabetes and hypertension in males could be involved [11].

The findings of a negative association between eGFR and BMI is consistent with some previous studies [19], but not all [20]. However, in contrast to the result that there was no significant association between BMI and eGFR in females, a study of 1878 people, aged between 19 and 77 years, found that obese women had a significantly reduced eGFR compared to non-obese women, but no association was found in men [18]. Other studies have suggested that obesity is associated with an increased eGFR, due to hyperfiltration [39]–[40]. However, only one of these studies involved adults over 60 years old [40], therefore, perhaps the initial increase in eGFR seen with an increased BMI is a compensatory mechanism, which later causes a decline in eGFR. This is supported by other studies, which have reported an increased BMI to be negatively associated with eGFR in older adults [16]–[17].

There have been conflicting reports into the effect of smoking on eGFR. In agreement with the results of this study, a number of studies have reported that cigarette smoking is associated with higher eGFR [22]–[24], while others have found the reverse [25], [41].

No associations with eGFR were found for SES at birth or at age 50 years. While previous studies have shown associations between SES in early life and later diseases such as cardiovascular disease [42] and in this cohort, associations with blood pressure [37], bone mineral density [43] and lung function [44], no studies have reported such associations with later kidney function. In contrast, previous studies have shown associations between adult kidney function and contemporary SES [30]–[31]. Similarly, no association was seen between eGFR and duration breast-fed. While formula-fed infants have been shown to have an increased kidney size compared to those were exclusively breastfed until 3 months of age [45], by 15 months of age, when all the children were on a standard diet, no difference in kidney size was observed.

### Strengths and limitations

The Newcastle Thousand Families study allows a large collection of prospective data to be analysed, and provides a unique opportunity to compare early and later life influences of kidney function at age 63–64 years. While the sample size of 354 participants was relatively small when compared to previous studies examining birth weight [11], SES [26], smoking [21] or sex and BMI [18] in relation to eGFR, a number of significant associations were shown in this study with the ability to assess them all simultaneously. Furthermore, the sample size was increased and made more representative by not limiting the study to those who still lived in Newcastle upon Tyne.

When examining the representativeness of the study in comparison to the original cohort, it was found that sex and SES at birth were both significantly different when compared to the proportions of the original cohort. No interactions were found for sex and variables significantly associated with eGFR, which confirms that there was no selection bias due to the change in representativeness of sex. Also, males and females were examined separately as well as combined. SES at birth was not found to be significantly associated with eGFR at age 63–64 years, but this cannot be representative of the original cohort, due to the significant change in the proportion of participants who returned from each category. Loss of oestrogen due to the menopause is potentially of a greater contribution to kidney function in females than the variables tested in this study. However, oestrogen levels were not assessed in this study, so further research into this hypothesis would be needed.

A further weakness when examining size at birth in comparison to eGFR is that data were only recorded for birth weight, rather than other indicators for size at birth, such as birth length, ponderal index and fetal weight-to-placental weight ratio index. However, standardised birth weight was used, which is a better measure of fetal growth than crude birth weight, but no association with eGFR was seen.

While for many years, eGFR has been used in assessing renal function, it has been shown to not be consistently associated with measured GFR across the normal range [46]. How this may impact on the research findings in this and other studies remains to be seen. Renal disease urine protein was not measured as part of this study, so we are not able to assess whether this would add to the hyperfiltration hypothesis.

## Conclusion

Birth weight and sex were the only early life factors found to be significantly associated with eGFR. However, no significant association was found for standardised birth weight. The contribution to eGFR from each of the significant variables was relatively small. The largest contribution to eGFR was from BMI, with nearly twice the variation explained than due to birth weight. As only a small amount of the variance in eGFR was accounted for by the variables investigated in this study, further studies comparing early and later life factors would be beneficial to determine different factors which may influence kidney function and which are the most important for focussing intervention studies.
